# Supplementary material for: Crawling and Gliding: A Computational Model for Shape-Driven Cell Migration
Source: PLoS Comput Biol. 2015 Oct 21;11(10):e1004280. doi: 10.1371/journal.pcbi.1004280 (PMC4619082; doi:10.1371/journal.pcbi.1004280)
Supplement: S1 Code — (ZIP) [file pcbi.1004280.s012.zip › release/tst/doc/html/random_8h.html]

Tissue Simulation Toolkit: random.h File Reference


Tissue Simulation Toolkit
 0.1.4.1


- Main Page
- Namespaces
- Classes
- Files

- File List
- File Members

Macros |
Functions

random.h File Reference

This graph shows which files directly or indirectly include this file:

Go to the source code of this file.

|  |  |
| --- | --- |
| Macros | |
| #define | MBIG   1000000000 |
|  | |
| #define | MSEED   161803398 |
|  | |
| #define | MZ   0 |
|  | |
| #define | FAC   (1.0/MBIG) |
|  | |

|  |  |
| --- | --- |
| Functions | |
| double | RANDOM () |
|  | |
| int | Seed (int seed) |
|  | |
| long | RandomNumber (long max) |
|  | |
| void | AskSeed () |
|  | |
| int | Randomize (void) |
|  | |

## Macro Definition Documentation

#define FAC   (1.0/MBIG)

Referenced by RANDOM().

#define MBIG   1000000000

Referenced by RANDOM().

#define MSEED   161803398

Referenced by RANDOM().

#define MZ   0

Referenced by RANDOM().

## Function Documentation

|  |  |  |  |  |  |
| --- | --- | --- | --- | --- | --- |
| void AskSeed | ( | void |  | ) |  |

Interactively ask for the seed

Parameters
:   |  |  |
    | --- | --- |
    | void |  |

Returns
:   void

References Seed().

|  |  |  |  |  |  |
| --- | --- | --- | --- | --- | --- |
| double RANDOM | ( | void |  | ) |  |

Returns
:   A random double between 0 and 1

|  |  |  |  |  |  |
| --- | --- | --- | --- | --- | --- |
| int Randomize | ( | void |  | ) |  |

Make a random seed based on the local time

Parameters
:   |  |  |
    | --- | --- |
    | void |  |

Returns
:   void

References Seed().

Referenced by Seed().

|  |  |  |  |  |  |
| --- | --- | --- | --- | --- | --- |
| long RandomNumber | ( | long | *max* | ) |  |

Returns a random integer value between 1 and 'max'

Parameters
:   |  |  |
    | --- | --- |
    | The | maximum value (long) |

Returns
:   A random integer (long)

References RANDOM().

Referenced by CellularPotts::GrowInCells(), CellularPotts::SetRandomTypes(), and CellularPotts::ThrowInCells().

|  |  |  |  |  |  |
| --- | --- | --- | --- | --- | --- |
| int Seed | ( | int | *seed* | ) |  |

Parameters
:   |  |  |
    | --- | --- |
    | An | integer random seed |

Returns
:   the random seed

References RANDOM(), and Randomize().

Referenced by AskSeed(), and Randomize().


---

Generated on Thu Aug 14 2014 22:04:01 for Tissue Simulation Toolkit by  

 1.8.6
